# Parasite transmission between trophic levels stabilizes predator–prey interaction

**DOI:** 10.1038/s41598-018-30818-7

**Published:** 2018-08-16

**Authors:** Akiyoshi Rogawa, Shigeki Ogata, Akihiko Mougi

**Affiliations:** Department of Biological Science, Faculty of Life and Environmental Science, Shimane University, 1060 Nishikawatsu-cho, Matsue, 690-8504 Japan

**Keywords:** Community ecology, Theoretical ecology

## Abstract

Manipulative parasites that promote their transmission by altering their host’s phenotype are widespread in nature, which suggests that host manipulation allows the permanent coexistence of the host with the parasite. However, the underlying mechanism by which host manipulation affects community stability remains unelucidated. Here, using a mathematical model, we show that host manipulation can stabilise community dynamics. We consider systems wherein parasites are transmitted between different trophic levels: intermediate host prey and final host predator. Without host manipulation, the non-manipulative parasite can destabilise an otherwise globally stable prey–predator system, causing population cycles. However, host manipulation can dampen such population cycles, particularly when the manipulation is strong. This finding suggests that host manipulation is a consequence of self-organized behavior of the parasite populations that allows permanent coexistence with the hosts and plays a key role in community stability.

## Introduction

Parasites are widespread in natural ecosystems and account for a significant proportion of the total biomass on earth^1,2^. They play critical roles in epidemiology and parasitology as well as in community structures, community dynamics and ecosystem functioning^3–5^.

Host manipulation, in which a parasite induces phenotypic changes of its host that promotes its own transmission^6,7^, has recently attracted attention from ecologists because of its prevalence across many phyla of protozoan and metazoan parasites^6^ and its potential to have a major impact on species interactions and community dynamics^8^. Such manipulation is expected to make differences between uninfected and infected individuals within a host population in terms of behaviours, physiology and life-history traits. This changes the intra- and interspecific interactions in the host species, which results in alterations in the structures and dynamics of the community^8^.

Although host manipulation would inherently have a major effect on community dynamics, how it influences community stability is poorly understood. A few reports have demonstrated that host manipulation drives population oscillations with large amplitudes, resulting in community instability^9,10^. However, in natural ecosystems, there are many examples of manipulative parasites permanently coexisting with their hosts^6,9^, which needs to be explained.

Herein, we propose a theory that explains the stable coexistence of manipulative parasites with their hosts. We consider two different systems in which parasites are transmitted between different trophic levels: intermediate host prey and final host predator. The first is intermediate host manipulation (IHM), in which a parasite manipulates the intermediate host in order to promote its transmission to the final host predator. For example, the invasion by conspicuous broodsacs of *Leucochloridium* spp. sporocysts into the tentacles of their intermediate terrestrial snail hosts would increase the likelihood of transmission to avian hosts by making the broodsacs more visible and more accessible to the predators. Moreover, by consuming the parasite’s eggs in avian faeces, the snail is again infected with the parasite^6,11^. The second type of system is final host manipulation (FHM), in which a parasite manipulates the final host in order to promote its transmission to the intermediate host prey. For example, nematomorpha, also known as horsehair worms, manipulate their final hosts cricket or mantis to enter streams, where the parasites reproduce and transmit their larvae to the intermediate host. By consuming the adults of the intermediate host prey, the final host predator is again infected with the parasite^12–15^.

In the present study, we develop a community dynamics model with these two major types of host manipulation (Methods). In IHM, the intermediate host prey is manipulated by the parasite so as to increase the risk of predation by the final host predator^16–19^. In FHM, the final host predator is manipulated in a way that induces its mortality, which provides the parasite with the opportunity to reproduce^15,20^. Here, we introduce a parameter, manipulation level, *m*. In IHM, we assume *a*_*u*_ < *a*_*i*_ = *ma*_*u*_, where *a*_*u*_ and *a*_*i*_ are the capture rates of uninfected and infected intermediate host prey by the final host predator. In FHM, we assume *c*_*u*_ < *c*_*i*_ = *mc*_*u*_, where *c*_*u*_ and *c*_*i*_ are the death rates of uninfected and infected final host predator. In addition, in both systems, the manipulated infected host (infected intermediate host prey in IHM and infected final host predator in FHM) is assumed to be infertile. In IHM and FHM, the parasite transmission from final host predator to intermediate host prey depends on the faeces and death of the final host predator, respectively (Methods). In each system, the other traits are same between infected and uninfected hosts.

In this study, we aim to examine the stability of coexistence of a manipulative parasite and the hosts, prey and the predator. Here, we define the stability as “converging towards a coexisting equilibrium”. The present model shows that, without host manipulation, the parasite can destabilise the otherwise globally stable prey–predator community. However, host manipulation can mitigate such potential instability, resulting in the stable coexistence of hosts with parasites. The present results suggest that host manipulation is a consequence of self-organized behavior of the parasite populations allowing parasites to permanently coexist with their hosts.

## Results

**Figure 1:**
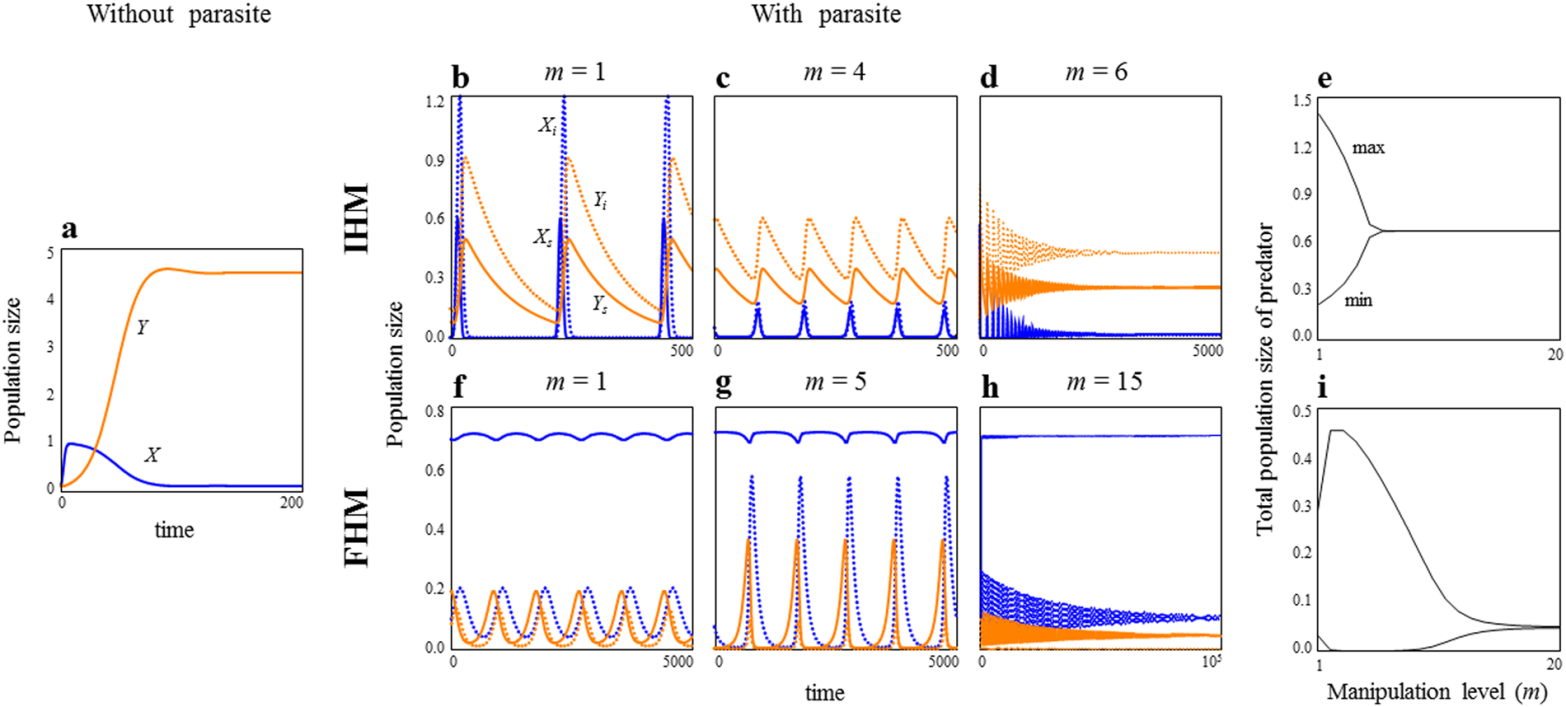
Dynamics of population sizes with varying host manipulation levels. (**a**) Population dynamics without parasite. Population dynamics in IHM and FHM are shown in (**b**–**d**) and (**f**–**h**), respectively. Solid blue, dotted blue, solid orange and dotted orange lines in (**b**–**d**) and (**f**–**h**) represent *X*_*u*_, *X*_*i*_, *Y*_*u*_, and *Y*_*i*_, respectively. In (**e**) and (**i**), bifurcation diagrams of population dynamics (predator) are plotted. Parameter values are: *b*_*u*_ = 1.05, *d*_*u*_ = 0.05, *ε* = 1, *v* = 2, *β*_*X*_ = *β*_*Y*_ = 1, *b*_*i*_ = 0, *d*_*i*_ = 0.05, *g*_*u*_ = *g*_*i*_ = 0.5, *c*_*u*_ = *c*_*i*_ = 0.01 and *a*_*u*_ = 0.2 in IHM; *b*_*u*_ = 1.01, *d*_*u*_ = 0.01, *b*_*i*_ = 1.01, *d*_*i*_ = 0.01, *ε* = 1.4, *v* = 2, *β*_*X*_ = *β*_*Y*_ = 1.5, *g*_*u*_ = 0.5, *g*_*i*_ = 0, *c*_*u*_ = 0.025 and *a*_*u*_ = *a*_*i*_ = 0.1 in FHM. Parameter values in (**a**) are the same as those in IHM except for *v* = 0. See Tables S1 and S2 for the details of parameter definitions and proposed values.

Without a parasite, the prey–predator system is always globally stable^21^ (Fig. 1a). However, once the parasite is introduced into the system, the parasite can destabilise the systems if the parasite is a non-manipulator (*m* = 1) (Fig. 1b,f). However, this is no longer true if the parasite strongly manipulates a host. The host manipulation mitigates population oscillations, and more powerful manipulation leads to stable coexistence in both IHM and FHM (Fig. 1d,e,h,i).

### Intermediate Host Manipulation

**Figure 2:**
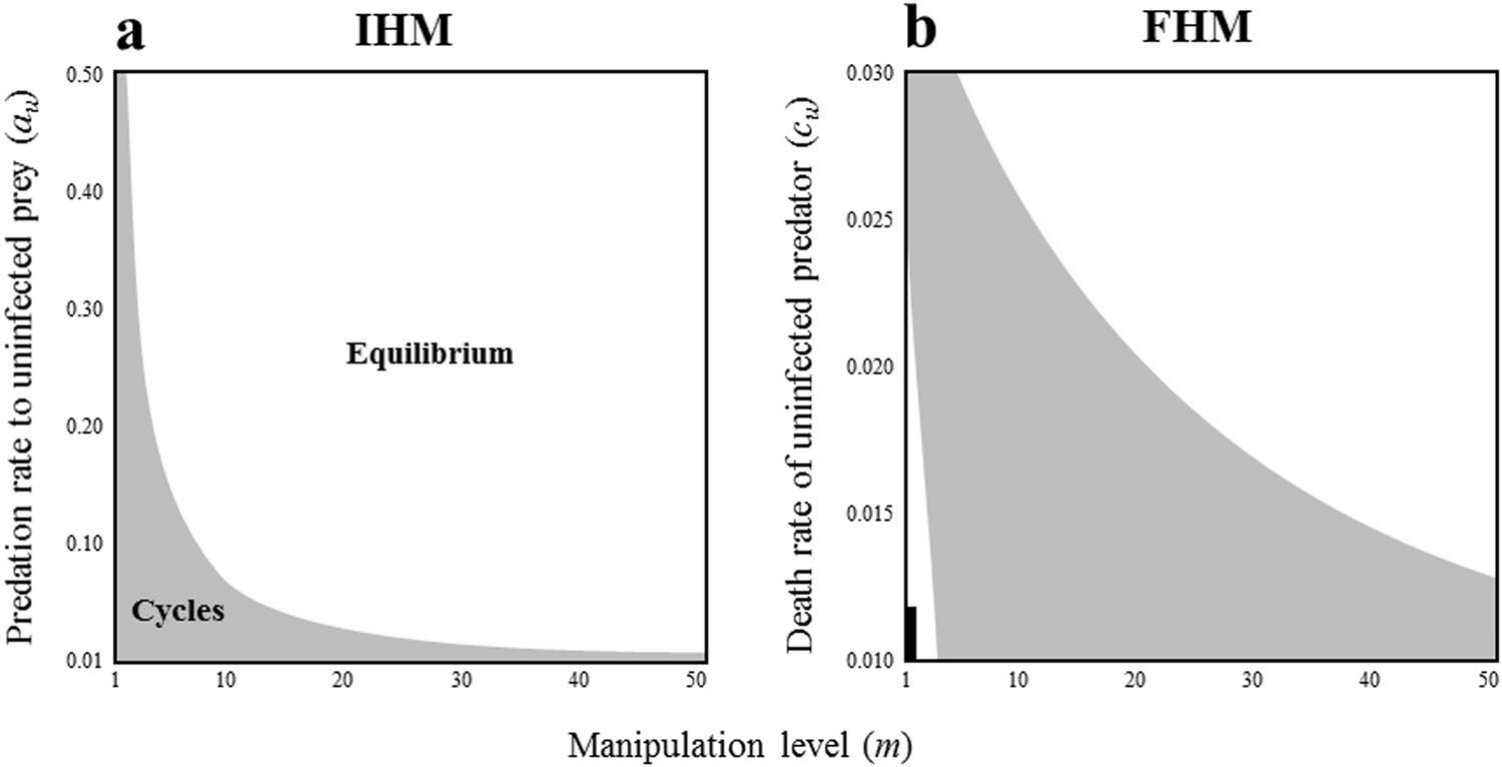
The relationship between host manipulation and local stability of the equilibrium in IHM (**a**) and FHM (**b**). Within the grey and white regions, the non-trivial equilibrium is locally unstable and stable, respectively. The black region represents trivial equilibrium. The stability is evaluated by the sign of a real part of dominant eigenvalues of Jacobian matrix. Parameters are the same as in Fig. 1.

**Figure 3:**
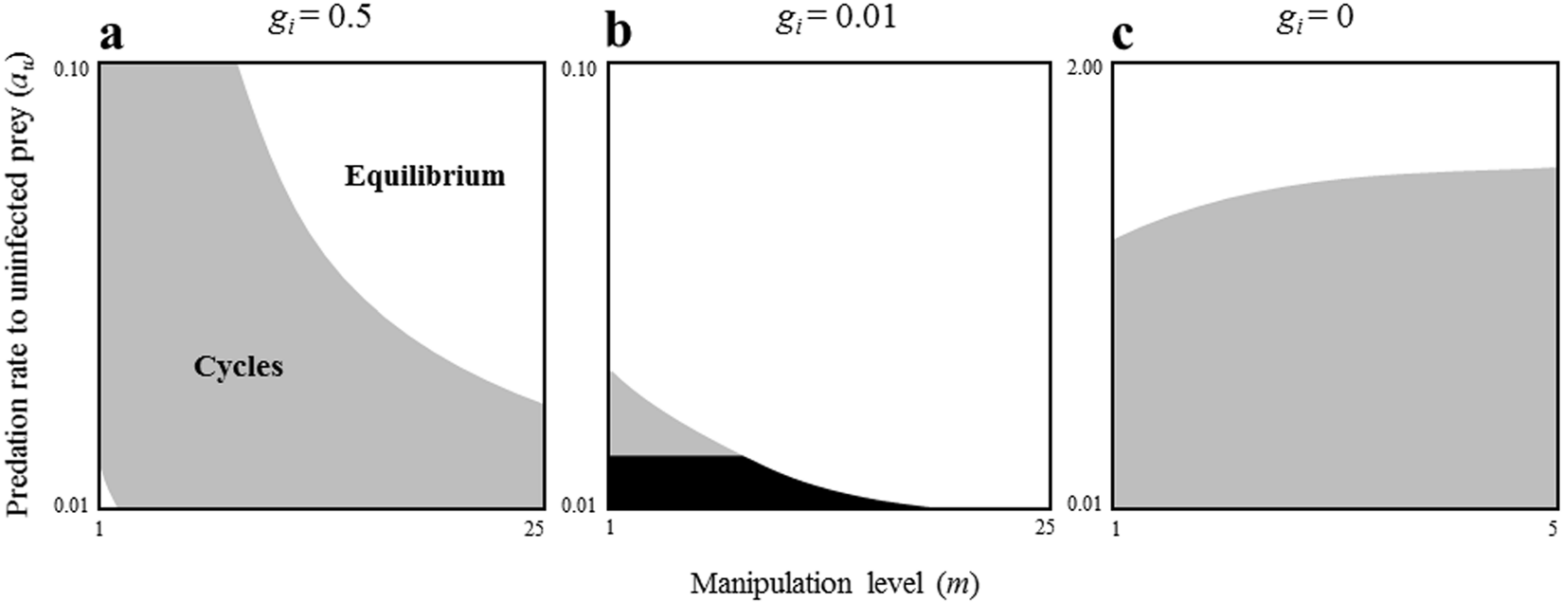
The relationship between host manipulation and local stability of the equilibrium in IHM varying with *g*_*i*_. (**a**) No effect of infection to fertility of infected host predator (*g*_*i*_ = *g*_*u*_). (**b**) Low fertility of infected host predator (*g*_*i*_ ≪ *g*_*u*_). (**c**) Infertility of infected host predator (*g*_*i*_ = 0). In (**a**), the parameter setting is same as Fig. 2a. Other information is the same as in Fig. 2a.

In IHM, if the predation rate of the infected intermediate host prey is larger than for the uninfected one and beyond a certain threshold, stable coexistence can occur (Fig. 2a). However, we find that whether the infected final host predator is infertile or not can critically affect the final outcome of the system (Fig. 3). When the infected final host predator cannot reproduce, the effect of manipulation on stability is completely reversed, namely, the manipulation destabilizes the system (Fig. 3c). Note that the manipulation can have a stabilising influence even if the reproduction rate of the infected final host predator is very small (Fig. 3b).

### Final Host Manipulation

In FHM, the effect of host manipulation on the stability of equilibrium depends on the death rates of final host predators (Fig. 2b). When the death rates of final host predators are high, host manipulation is likely to lead to stable coexistence. In contrast, when the death rates of final host are low, incomplete manipulation can destabilize the system, although such instability can be stabilized again by stronger manipulation (Fig. 2b). Contrary to IHM, in FHM, whether the infected intermediate host prey is infertile or not does not affect the result (Fig. S1).

These results suggest that host manipulation can have a stabilising effect on the community dynamics of the parasite’s hosts, namely, both prey and predator. This tendency does not depend on whether the manipulated host is prey or predator. Further, the choice of parameters does not qualitatively change the effect of host manipulation (Figs S2 and S3).

## Discussion

The present study showed that a manipulative parasite can stabilise the population dynamics of its hosts, both prey and predator. Without host manipulation, the parasite can induce population oscillations in otherwise stable prey–predator systems. Host manipulation can act as a stabiliser of community dynamics, particularly when the manipulation is more intense. These results suggest that host manipulation is a consequence of self-organized behavior of the parasite populations allowing the parasite to permanently coexist with its hosts, and plays a major role in community stability.

### Intermediate Host Manipulation

Previous theoretical studies demonstrated that host manipulation tends to destabilise the community dynamics^9,10^, contrary to our prediction. In one of these studies, a system quite similar to the IHM system was proposed. However, the infected final host predator was assumed to be infertile, contrary to our model^10^. The present study showed that this assumption critically affects the result (Fig. 3). The manipulation shifts to a stabilising role if the infected final host predator can reproduce. These opposite outcomes are explained by the following mechanisms. If the infected host predator cannot reproduce (previous model), an increase in host predator abundance is suppressed because infected host predator increased by host manipulation does not contribute to predator growth. This causes a delay in the suppression of prey growth, resulting in population oscillations. However, if the infected host predator can reproduce (our model), the situation completely changes. Because the host manipulation does not reduce the suppression of prey growth, the predator–prey negative feedback^21^ normally operates, stabilizing the system. Hence, reproduction of infected host predators is necessary for host manipulation to play a stabilizing role in prey–predator dynamics. This suggests that parasites manipulating intermediate host prey should not affect the ability of the final host predator to reproduce. Another study also considered an IHM system without assuming the infertility of the infected hosts, with density-dependent self-regulation in infected prey and a frequency-dependent functional response of predation^9^. Since the frequency-dependent predation should hinder the effect of host manipulation, it would not prevent the parasite-induced oscillation.

The manipulative parasites can castrate the intermediate host prey^22,23^. For example, a manipulative parasite, *Tokoplasma*, makes the intermediate host prey, mice, infertile^24^. A trematode flatworm parasite also castrates the intermediate host prey mud snail^22^. In contrast, to our knowledge, we have no evidence that shows such castrating effect on the infected individuals of final host predators. Also, even if manipulation does not cause castration of intermediate host prey, it would decrease the chance of reproduction^23^ through changing a niche, suffering at some cost, and/or increasing the risk of predation from the final host predator^25,26^. It is important to systematically compare the reproductive abilities of uninfected and infected individuals of different hosts in order to confirm our model assumption.

### Final Host Manipulation

A similar stabilisation mechanism also acts in FHM. By killing or reducing the number of infected infertile final host predators through host manipulation, the suppression of the total number of prey due to uninfected predator can be maintained. This stabilising effect is predicted to be stronger as the level of manipulation increases. Such powerful manipulation might actually occur in the wild. For example, infected crickets, which can be an important seasonal prey subsidy, accounted for 60% of the annual energy intake of an endangered Kirikuchi char population, *Salvelinus leucomaenis japonicus*, in a temperate Japanese stream^15^. Surprisingly, the infected crickets were 20 times more likely to fall into streams than the uninfected ones. This manipulation can be so powerful as to cause dramatic change of the aquatic ecosystem^27^. In addition, as with IHM, a parasite, horsehair worm, castrates the cricket (horsehair worms matured in a cricket consumes reproductive organs of the cricket)^28,29^. However, we have no evidence that shows some castrating effect on the intermediate host prey, such as aquatic invertebrates that move on land after metamorphosis.

In the present study, the functional response of predators to prey density has been assumed to be linear for simplicity. However, we can also show that relaxation of this simplification does not affect the stabilizing effect of host manipulation. In a non-linear functional response, the system can exhibit population oscillations even without a parasite^30^. However, a strong host manipulation by parasite can mitigate such prey–predator oscillation in both IHM and FHM (Fig. S4). This suggests a robustness of the stabilizing role of host manipulation in predator–prey interaction.

Host manipulation is an adaptive strategy promoting transmission to the next host. Trophically transmitted parasite systems in natural ecosystems might be stabilised by its adaptation. In fact, it is suggested that a manipulative parasite tapeworm can contribute to the coexistence of hosts predator wolf and prey moose^29^. However, because the ecological population dynamics and evolutionary dynamics of traits can interact with each other, it will be necessary to study the eco-evolutionary dynamics^31^ of a manipulative parasite and its hosts. The evolution of multiple traits including infection rates associated with manipulation and counter-evolution of hosts are also important challenges.

## Methods

Consider a prey–predator system with a manipulative parasite. The parasites can transmit to the final host predator through predation of the infected-intermediate host prey. It might reproduce by escaping the final host body or through defecation of the host. The intermediate host prey is parasitized through occasional intrusion of free-living parasites into the body, or contacting and/or eating faeces. A general model of such a complex life cycle of the parasite is described by the following differential equations:

1a $$\frac{d{X}_{u}}{dt}=({b}_{u}-{d}_{u}){X}_{u}+{b}_{i}{X}_{i}-\varepsilon ({X}_{u}+{X}_{i}){X}_{u}-{a}_{u}({Y}_{u}+{Y}_{i}){X}_{u}-{\beta }_{X}f({X}_{u},{Y}_{i}),$$

1b $$\frac{d{X}_{i}}{dt}={\beta }_{X}f({X}_{u},{Y}_{i})-{d}_{i}{X}_{i}-{a}_{i}({Y}_{u}+{Y}_{i}){X}_{i},$$

1c $$\frac{d{Y}_{u}}{dt}={g}_{u}{a}_{u}({X}_{u}+{X}_{i}){Y}_{u}+{g}_{i}{a}_{i}({X}_{u}+{X}_{i}){Y}_{i}-{c}_{u}{Y}_{u}-{\beta }_{Y}{a}_{i}{X}_{i}{Y}_{u},$$

1d $$\frac{d{Y}_{i}}{dt}={\beta }_{Y}{a}_{i}{X}_{i}{Y}_{u}-{c}_{i}{Y}_{i},$$

where *X*_*u*_, *X*_*i*_, *Y*_*u*_ and *Y*_*i*_ represent the population sizes (or densities) of the uninfected intermediate host prey, infected intermediate host prey, uninfected final host predator, and infected final host predator, respectively. *b*_*j*_ (*j* = *u* or *i*) are the birth rates of the uninfected and infected prey; *d*_*j*_ (*j* = *u* or *i*) are the death rates of the uninfected and infected prey; *ε* is the self-regulation coefficient of the prey; *a*_*j*_ (*j* = *u* or *i*) are the capture rates of uninfected and infected prey by the predator, defined as the per capita rate at which a predator captures the prey; *g*_*j*_ (*j* = *u* or *i*) are the conversion efficiency, which relates to the birth rate of the predator to its prey consumption; *β*_*X*_ and *β*_*Y*_ are the infection rate of parasite to prey and predator; and *c*_*j*_ (*j* = *u* or *i*) are the death rates of the uninfected predator and infected predator, respectively.

We focus on two major types of host manipulation: (i) intermediate host manipulation (IHM) and (ii) final host manipulation (FHM). In IHM, the only intermediate host is manipulated by the parasite in a way that increases predation to the final host predator (*a*_*u*_ < *a*_*i*_ = *ma*_*u*_, where *m* is the manipulation level). When *m* = 1, the parasite has no manipulation effect. We also assume that the infection makes intermediate host infertile (*b*_*i*_ = 0), and cause the infected host to occupy a different niche, resulting in the elimination of competition among uninfected and infected individuals within the host (*εX*_*i*_ = 0)^26,32^. Because we assume the parasite manipulates the only intermediate host prey, the characteristics of uninfected and infected final hosts are assumed to be the same (*c*_*j*_ = *c*_*u*_, *g*_*i*_ = *g*_*u*_). The functional form of *β*_*X*_*f*(*X*_*u*_, *Y*_*i*_) is *β*_*X*_*X*_*u*_*vY*_*i*_ where *v* is the reproduction rate of parasites (which also might be related to the excretion rate of the predator). At rate *v*, parasites are produced by infected final host predator *Y*_*i*_ and passed into the environment, where they encounter (and transmit to) the intermediate host at rate *β*_*X*_*X*_*u*_.

In FHM, the only final host is manipulated by the parasite in a way that allows parasite reproduction through killing of the host (*c*_*u*_ < *c*_*i*_ = *mc*_*u*_, where *m* is the manipulation level). We also assume that the infection makes final host infertile (*g*_*i*_ = 0). The functional form of *β*_*X*_*f*(*X*_*u*_, *Y*_*i*_) is *β*_*X*_*X*_*u*_*vc*_*i*_*Y*_*i*,_ where *v* is the reproduction rate of parasites. The parasites escaped from dead infected final host predator *c*_*i*_*Y*_*i*_ reproduce at rate *v*. They encounter (and transmit to) the intermediate host at rate *β*_*X*_*X*_*u*_. The characteristics of uninfected and infected intermediate hosts are assumed to be the same (*b*_*i*_ = *b*_*u*_, *d*_*i*_ = *d*_*u*_ and *a*_*i*_ = *a*_*u*_).

Under the two scenarios (i and ii), by setting the right-hand sides of Eqs 1a–d to zero, each non-trivial equilibrium (*X*_u_*, *X*_i_*, *Y*_u_*, *Y*_i_*) is obtained as:

i $$(\frac{{r}_{u}-{a}_{u}{Y}^{\ast }-{\beta }_{X}v{{Y}_{i}}^{\ast }}{\varepsilon },{\beta }_{X}v{{X}_{u}}^{\ast }{{Y}_{i}}^{\ast }({d}_{i}+{a}_{i}{Y}^{\ast }),\frac{g({a}_{u}{{X}_{u}}^{\ast }+{a}_{i}{{X}_{i}}^{\ast }){{Y}_{i}}^{\ast }}{c-g{a}_{u}{{X}_{u}}^{\ast }-(g-{\beta }_{Y}){a}_{i}{{X}_{i}}^{\ast }},\frac{{\beta }_{Y}{a}_{i}{{X}_{i}}^{\ast }{{Y}_{u}}^{\ast }}{c})$$

ii $$(\frac{{{X}_{i}}^{\ast }({d}_{i}+{a}_{i}{Y}^{\ast })}{{\beta }_{X}v{c}_{i}{{Y}_{i}}^{\ast }},\frac{{c}_{u}\mbox{--}{g}_{u}{a}_{u}{{X}_{u}}^{\ast }}{({g}_{u}\mbox{--}{\beta }_{Y}){a}_{i}},\frac{{r}_{u}{{X}_{u}}^{\ast }+{b}_{i}{{X}_{i}}^{\ast }}{{a}_{u}{{X}_{u}}^{\ast }{{X}_{u}}^{\ast }}+\frac{({a}_{u}+{\beta }_{X}v{c}_{i}){{Y}_{i}}^{\ast }+\varepsilon {X}^{\ast }}{{a}_{u}},\frac{{\beta }_{Y}{a}_{i}{{X}_{i}}^{\ast }{{Y}_{u}}^{\ast }}{{c}_{i}})$$

where *c* = *c*_*u*_ = *c*_*i*_, *r*_*u*_ = *b*_*u*_ – *d*_*u*_, *g* = *g*_*u*_ = *g*_*i*_, X* = *X*_u_^*^ + *X*_i_^*^ and Y* = *Y*_u_^*^ + *Y*_i_^*^. Note that the equilibrium in (i) has an explicit formulation (not shown to avoid complexity), while that in (ii) does not (but does if *b*_*i*_ = 0 and *εX*_*i*_ = 0).

By using local stability analysis, the stability of the coexistence equilibria can be numerically examined. We can judge the local stability by the sign of a real part of the dominant eigenvalue of the Jacobian matrix (negative is stable).

## Electronic supplementary material

Supplemental figures
