# Author Correction: Parasite transmission between trophic levels stabilizes predator–prey interaction

**DOI:** 10.1038/s41598-019-49487-1

**Published:** 2019-10-15

**Authors:** Akiyoshi Rogawa, Shigeki Ogata, Akihiko Mougi

**Affiliations:** Department of Biological Science, Faculty of Life and Environmental Science, Shimane University, 1060 Nishikawatsu-cho, Matsue, 690-8504 Japan

Correction to: *Scientific Reports* 10.1038/s41598-018-30818-7, published online 16 August 2018

This Article contains an error in Equation 1c, where

1c $$\frac{d{Y}_{u}}{dt}={g}_{u}{a}_{u}({X}_{u}+{X}_{i}){Y}_{u}+{g}_{i}{a}_{i}({X}_{u}+{X}_{i}){Y}_{i}-{c}_{u}{Y}_{u}-{\beta }_{Y}{a}_{i}{X}_{i}{Y}_{u},$$

should read:

1c $$\frac{d{Y}_{u}}{dt}={g}_{u}({a}_{u}{X}_{u}+{a}_{i}{X}_{i}){Y}_{u}+{g}_{i}({a}_{u}{X}_{u}+{a}_{i}{X}_{i}){Y}_{i}-{c}_{u}{Y}_{u}-{\beta }_{Y}{a}_{i}{X}_{i}{Y}_{u},$$

