# Supplementary material for: Parasite transmission between trophic levels stabilizes predator–prey interaction
Source: Sci Rep. 2018 Aug 16;8:12246. doi: 10.1038/s41598-018-30818-7 (PMC6095923; doi:10.1038/s41598-018-30818-7)
Supplement: Supplementary file 1 — Supplemental figures [file 41598_2018_30818_MOESM1_ESM.pdf]

Supplementary Information

Parasite transmission between trophic levels stabilizes predator-prey interaction

A. Rogawa, S. Ogata, A. Mougi

Supplemental Figures

Fig. S1. The relationship between host manipulation and local stability of the equilibrium in FHM. We assumed $b_i = 0$ and $\varepsilon X_i = 0$. Other information is the same as in Fig. 2a.

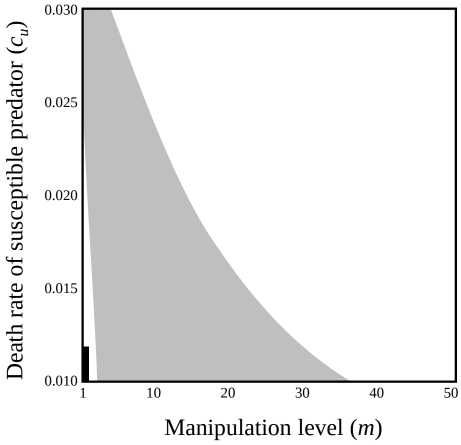

Fig. S2. The relationship between host manipulation and local stability of the equilibrium in IHM. In (a–f), the effects of parameters indicated at the top of panels are shown. $g = g_u = g_i$ and $c = c_u = c_i$. Other information is the same as in Fig. 2a.

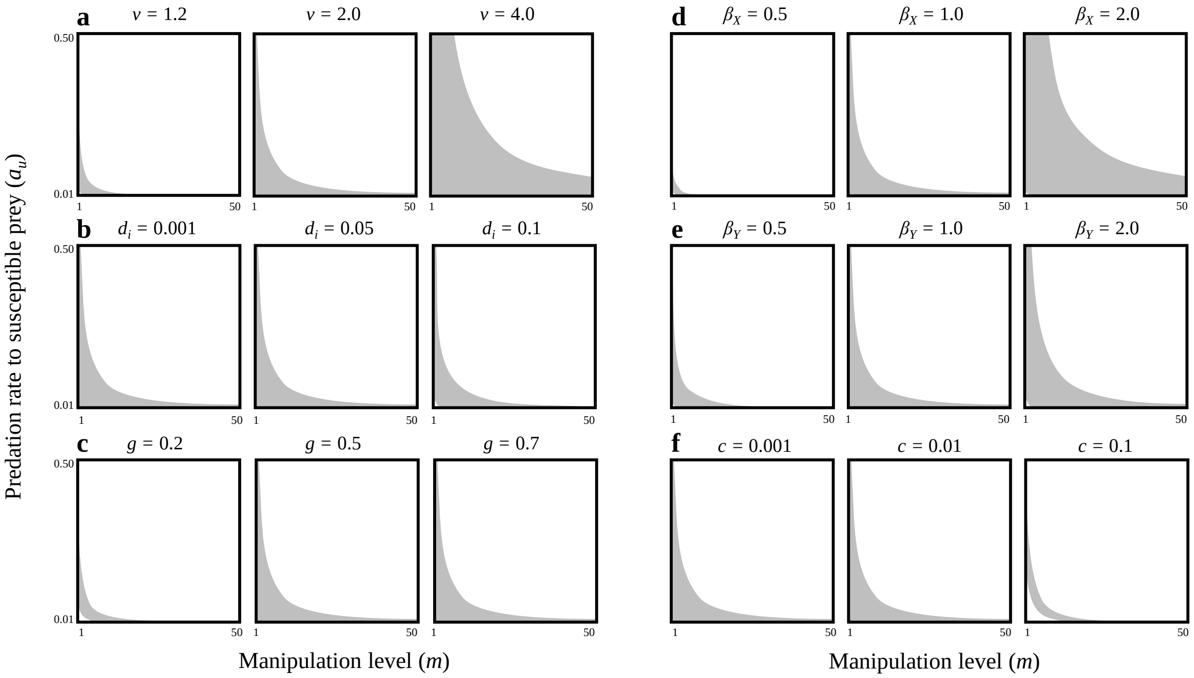

Fig. S3. The relationship between host manipulation and local stability of the equilibrium in FHM. In (a–f), the effects of parameters indicated at the top of panels are shown. $a = a_u = a_i$. Other information is the same as in Fig. 2b.

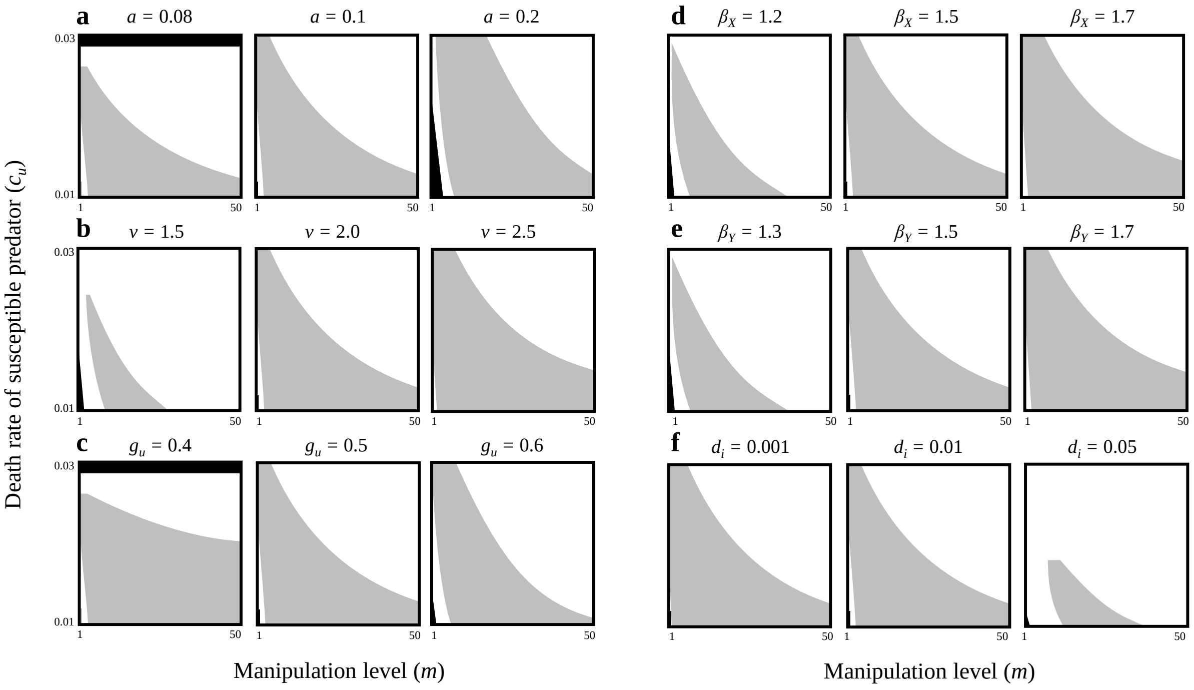

Fig. S4. Effects of host manipulation on the amplitude of population oscillation in IHM (a) and FHM (b). A non-linear functional response, $a_jX_j/(1+a_jh_jX_j)$ (where h is the prey handling time), is assumed. Within the shaded region, the parasite cannot persist. Parameters are chosen so as to be unstable even without a parasite. Parameter values are: b_u = 1.01, d_u = 0.01, ε = 1, h = 3, v = 2, $\beta_X = \beta_Y$ = 1, b_i = 0, d_i = 0.01, $g_u = g_i$ = 0.2, $c_u = c_i$ = 0.01 and a_u = 0.5 in IHM; b_u = 2.8, d_u = 1.8, b_i = 2.8, d_i = 1.8, ε = 1, v = 1.8, β_X = 4, β_Y = 2, g_u = 0.6, g_i = 0, c_u = 0.02 and $a_u = a_i$ = 1.1 in FHM.

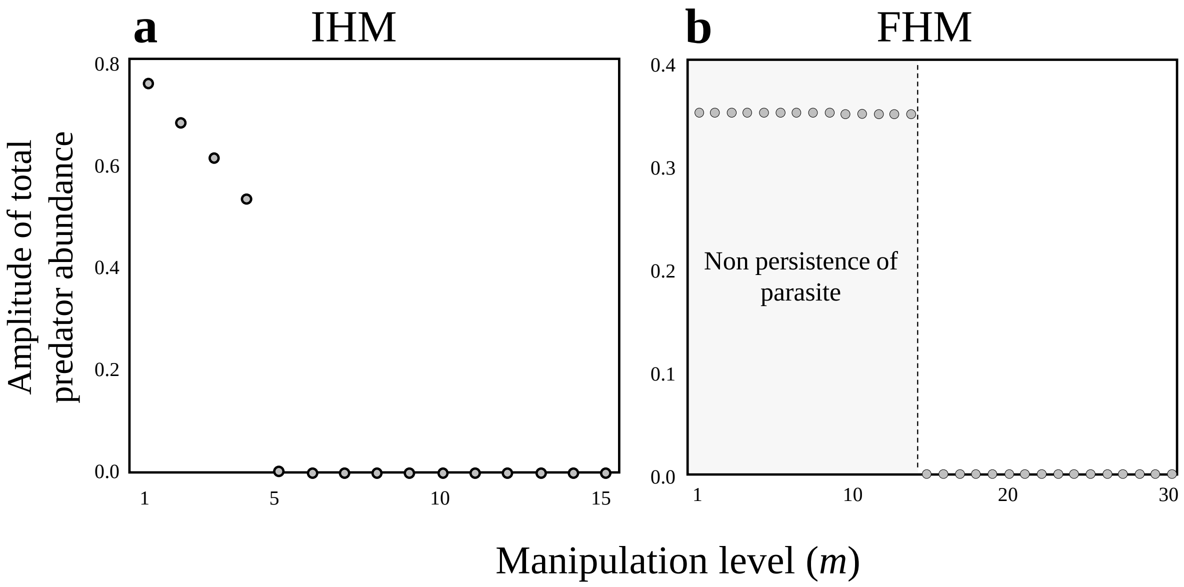

Supplemental Tables

Table S1. Parameter definitions and proposed values in IHM.

Symbol	Definition	Value
b_u	Birth rate of uninfected prey	1.05
b_i	Birth rate of infected prey	0
d_u	Death rate of uninfected prey	0.05
d_i	Death rate of infected prey	0.05
ε	Self-regulation coefficient of prey	1
a_u	Capture rate of uninfected host prey by predator	0.2
a_i	Capture rate of infected host prey by predator (= ma_u)	varied
g_u	Uninfected prey conversion efficiency of predator	0.5
g_i	Infected prey conversion efficiency of predator	0.5
c_u	Death rate of uninfected predator	0.01
c_i	Death rate of infected predator	0.01
β_X	Infection rate of parasite to prey	1
β_Y	Infection rate of parasite to predator	1
v	Reproduction rate of parasite	2
m	Manipulation level	varied

Table S2. Parameter definitions and proposed values in FHM.

Symbol	Definition	Value
b_u	Birth rate of uninfected prey	1.01
b_i	Birth rate of infected prey	1.01
d_u	Death rate of uninfected prey	0.01
d_i	Death rate of infected prey	0.01
ε	Self-regulation coefficient of prey	1.4
a_u	Capture rate of uninfected host prey by predator	0.1
a_i	Capture rate of infected host prey by predator	0.1
g_u	Uninfected prey conversion efficiency of predator	0.5
g_i	Infected prey conversion efficiency of predator	0
c_u	Death rate of uninfected predator	0.025
c_i	Death rate of infected predator (= mc_u)	varied
β_X	Infection rate of parasite to prey	1.5
β_Y	Infection rate of parasite to predator	1.5
v	Reproduction rate of parasite	2
m	Manipulation level	varied
